# Sustainable Electrolyte Media in Dye-Sensitized Solar Cells: From Water-Based to Deep Eutectic Solvents and Biopolymeric Approaches

**DOI:** 10.3390/molecules31122037

**Published:** 2026-06-10

**Authors:** Giorgia Salerno, Norberto Manfredi, Alessandro Abbotto, Ottavia Bettucci

**Affiliations:** Department of Materials Science, Solar Energy Research Centre MIB-SOLAR, INSTM Milano-Bicocca Research Unit, CINMPIS Consortium, University of Milano-Bicocca, Via Cozzi 55, 20125 Milano, Italy; giorgia.salerno@unimib.it (G.S.); norberto.manfredi@unimib.it (N.M.)

**Keywords:** sustainable DSSCs, DES, sustainable electrolytes

## Abstract

Dye-sensitized solar cells (DSSCs) represent a promising photovoltaic technology for indoor and building-integrated applications due to their colour tunability, semi-transparency, and favourable spectral response. However, the sustainability of conventional devices is hindered by the use of volatile organic solvent-based electrolytes, which raise concerns regarding toxicity, flammability, and long-term stability. This review analyses the evolution of DSSC architecture, with particular focus on electrolyte media, ranging from aqueous systems to deep eutectic solvents and bio-derived quasi-solid architectures. Special attention is focused on the interplay between electrolyte composition, dye design, and interfacial charge-transfer processes. By highlighting recent progress and remaining challenges, this work outlines viable strategies toward safe, durable, and fully sustainable DSSCs tailored for indoor and integrated photovoltaic applications.

## 1. Introduction

DSSCs have historically attracted attention for their potential as low-cost, flexible photovoltaic devices. While early research primarily focused on outdoor solar energy harvesting, it is now widely acknowledged that DSSCs are not intended to directly compete with crystalline silicon photovoltaics in terms of peak outdoor efficiency, particularly under direct sunlight [1]. Instead, contemporary studies highlight how the intrinsic strengths of DSSCs such as semi-transparency, colour tunability, and a favourable spectrum make them particularly suitable for building-integrated and indoor photovoltaics [2,3,4,5,6]. This shift in focus is further justified by the rapid growth of the Internet of Things (IoT) ecosystem, which demands autonomous power sources for billions of interconnected devices. In this context, DSSCs have emerged as a leading technology for low-irradiance environments; unlike traditional photovoltaics, their efficiency often increases under artificial indoor lighting (such as LEDs or fluorescent lamps), where they can effectively harvest energy to power sensors and low-power electronics. More specifically, DSSCs can be customized through the selection of organic dyes tailored to match the emission spectra of indoor light sources as well as the optical requirements of facade-integrated elements, thereby maximizing their overall energy-harvesting efficiency [7,8]. These peculiar features keep DSSCs highly attractive for niche applications compared to other PV technologies, offering a viable and environmentally conscious alternative to conventional devices in diffuse light conditions. Moreover, among all photovoltaic technologies, DSSCs stand out as a viable and environmentally conscious alternative to conventional photovoltaic devices [1]. However, to be considered sustainable throughout their entire lifecycle, DSSC components require simultaneous optimization. In particular, the electrolyte (typically a redox pair such as I^−^/I_3_^−^, Co^2+^/Co^3+^, or Cu^+^/Cu^2+^ dissolved in a liquid medium) [9,10,11] presents significant safety and environmental challenges, often being composed of volatile organic compounds (VOCs), thus raising concerns related to their toxicity, flammability, and atmospheric persistence.

Traditional electrolyte formulations are, indeed, predominantly based on acetonitrile (ACN) due to its low viscosity and high dielectric constant, which facilitate rapid ionic transport [12]. Despite their performance benefits, the replacement of these solvents is critical from a safety and sustainability perspective: ACN is not only highly flammable and volatile, leading to internal pressure buildup and potential leakage, but also poses significant toxicity risks to human health and the environment. Furthermore, its atmospheric persistence and the carbon footprint associated with its petrochemical synthesis conflict with the ‘green’ premise of next-generation photovoltaics.

All these factors affect the overall sustainability of DSSCs over their operational lifetime [13,14,15]. Moreover, outdoor DSSCs are exposed to UV radiation, temperature fluctuations, and humidity, which exacerbate volatility and redox instability, making traditional organic electrolytes less desirable unless sophisticated encapsulation is employed [16,17,18]. Indoor DSSCs, by contrast, operate under milder conditions with lower light intensity, which alleviates some stability constraints. However, safety considerations become even more stringent in indoor applications, where the presence of VOCs increases risks in the event of leakage or accidental damage in a domestic environment. Nevertheless, outdoor, indoor, and building-integrated applications increasingly demand eco-friendly, non-toxic, and esthetically compatible electrolytes, reflecting a growing emphasis on sustainability and safety in real-world deployment. For all these reasons, current research is increasingly directed toward developing innovative electrolyte media formulations and, consequently, novel architectures and/or dyes compatible with such electrolytes to make the technology greener and safer. Despite these efforts, it is not easy to design and replace traditional electrolytes, as they require key features, such as high charge mobility, adequate chemical and electrochemical stability, efficient redox-shuttle diffusion, and low viscosity, to perform effectively in the devices. Moreover, electrolytes play a crucial role in the device architecture in terms of electron transfer, dye regeneration kinetics, and sustained ionic transport [1,19,20,21].

**Figure 1:**
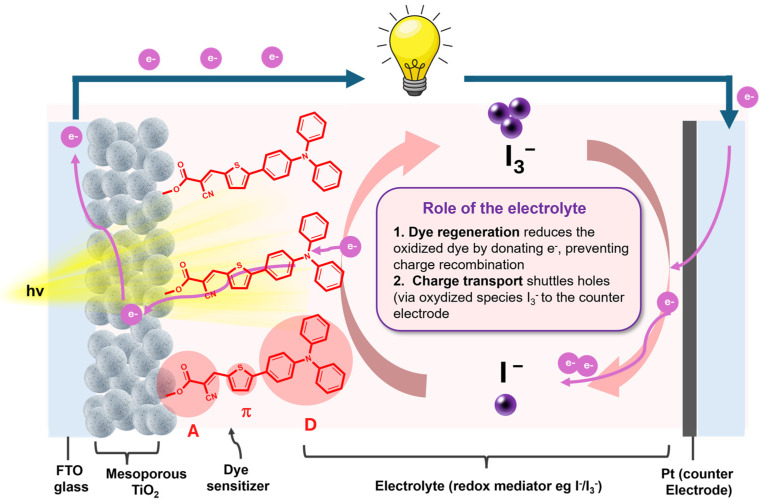
Working principles of a DSSC sensitized by a representative organic D-π-A dye (where π denotes a π-conjugated bridge connecting the donor (D) and acceptor (A) moieties of the sensitizer), and the role of the electrolyte redox couple.

In a DSSC, light absorption excites an electron from the dye’s HOMO (Highest Occupied Molecular Orbital) localized on the donor moiety (D) of the dye) to its LUMO (Lowest Unoccupied Molecular Orbital) localized on the acceptor moiety (A). The electron is then injected into the TiO_2_ conduction band and flows through the external circuit to the counter-electrode, generating photocurrent [22,23]. However, to maintain continuous operation, the oxidized dye must be rapidly regenerated (Figure 1). The electrolyte fulfils this critical role by donating an electron to the oxidized dye via its redox couple, thereby restoring the dye to its ground state. Simultaneously, the electrolyte mediates internal charge transport by shutting the oxidized species to the counter electrode, where they are reduced by electrons returning through the circuit (Figure 1) [21].

Thus, the electrolyte is essential not only for dye regeneration but also for maintaining ionic conductivity, charge neutrality, and efficient electron flow, making it a central component of DSSC performance and stability. Early efforts to replace VOCs as electrolyte media explore the use of ionic liquids (ILs) [24,25,26,27,28,29], gel polymer matrices (GPEs) [30,31,32,33], and supramolecular IL gels [34,35,36]. Although these electrolyte media improved thermal stability and minimized evaporation, they also suffered from significant drawbacks. ILs, for instance, have very high viscosity that severely limits the diffusion coefficient of the I^−^/I_3_^−^ redox couple, reducing dye regeneration rates [37,38]. Their synthesis can be costly, and some ILs raise concerns about toxicity or long-term chemical stability [39]. Meanwhile, GPEs offer better leakage resistance but typically exhibit lower ionic conductivity and mass transport limitations due to the polymer network, which can also degrade or interact unfavourably with electrolyte species over time [30,40]. In response to these challenges, research has increasingly focused on sustainable electrolyte media that combine safety, environmental compatibility, adequate ionic transport, and low costs.

In this review, alternative strategies to obtain safe and sustainable electrolyte formulations for DSSCs will be analyzed. Starting from the most studied water-based electrolytes, which are inherently non-toxic and environmentally friendly, although conductivity and stability remain challenging, the discussion will then move to deep eutectic solvent (DES)-based electrolytes, which are cheap, based on abundant materials, chemically tunable, low-volatile, and frequently described as sustainable alternatives to VOCs due to the potential non-toxicity and biodegradability of many common formulations (e.g., those based on choline chloride and natural hydrogen bond donors) [41,42]. Finally, the most innovative bio-derived quasi-solid architecture (e.g., hydrogels, polymer-based gels) capable of reducing leakage, enhancing mechanical robustness, and facilitating integration into flexible or confined geometries will be addressed.

## 2. Water-Based Electrolytes in DSSCs

**Figure 2:**
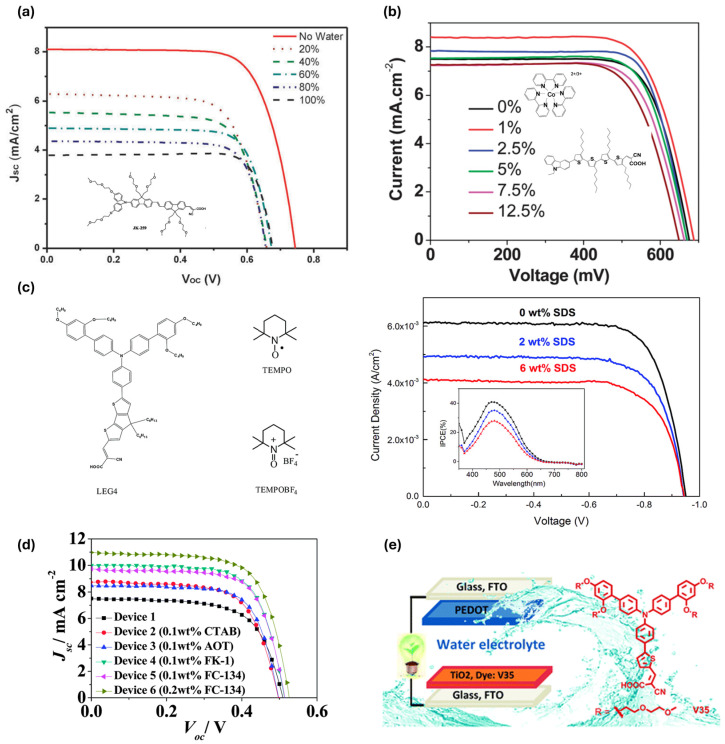
First attempts of aqueous DSSCs. (**a**) First rationalization of organic dye behaviours in aqueous-based electrolytes [48]; (**b**) use of Co(II/III) tris(2,2′-bipyridine) as a redox couple in aqueous media in place of I**^−^**/I**_3_^−^** systems [49]; (**c**) use of TEMPO/TEMPO**^+^** as redox couples in aqueous media [50]; (**d**) *J/V* curves of fully water-based devices with different surfactants to increase ionic conductivity and mitigate aggregation in water-based electrolytes [51]; (**e**) innovative hydrophilic organic dyes for water-based DSSCs [52].

In the landscape of sustainable DSSCs, the use of water as electrolyte media emerged as a promising strategy for developing environmentally friendly and intrinsically safe photovoltaic devices. Water-based electrolytes offer several advantages over conventional VOC formulations, including non-toxicity, low cost, and negligible volatility, making them particularly attractive for practical applications. The use of water, once considered impractical due to stability concerns (as it promotes hydrolysis of the ester-like linkage between the dye and the TiO_2_ surface), has gained attention over the past decade [43,44,45]. This challenge was primarily addressed through the development of hydrophobic sensitizers and alternative anchoring groups (e.g., hydroxamic acids or silyl groups) that create a more stable and water-resistant interface, preventing the nucleophilic attack of water molecules on the TiO_2_–dye linkage and thereby suppressing hydrolysis [46]. As explored in one of the first reviews on this topic by Bella et al. [47], water-based DSSCs can deliver interesting device functions (light absorption, electron injection, dye regeneration, and charge transport) while offering clear advantages in terms of cost, environmental impact, non-flammability, and reduced volatility. Among the pioneering studies in this field, O’Regan et al. reported a comprehensive investigation of water-based electrolytes in DSSCs, highlighting both the potential of fully aqueous systems and the critical challenges associated with charge recombination and dye/electrode stability in water [41]. A few years later, Ko et al. employed organic sensitizers in water-based electrolytes, demonstrating that conventional dyes can operate in water but often with lower efficiency due to limited solubility and recombination issues (Figure 2a) [48]. A complementary approach involving the use of Co(II/III) tris(2,2′-bipyridine) redox couples was explored in the same years providing faster electron transfer and reduced recombination compared to I^−^/I_3_^−^ systems, though at the cost of increased synthetic complexity (Figure 2b) [49]. Similarly, the TEMPO/TEMPO^+^ ((2,2,6,6-tetramethylpiperidin-1-yl) oxyl) redox couple has also been demonstrated to be efficient in aqueous media, offering rapid dye regeneration and improved photovoltage *V*_oc_, although challenges remain in maintaining long-term stability (Figure 2c) [50]. To further enhance the performance, the addition of ionic surfactants was explored revealing the capability to increase ionic conductivity and mitigate aggregation of dye molecules in aqueous environments (Figure 2d) [51]. On this account, studies on hydrophilic organic dyes were also developed specifically for water-based DSSCs, improving light absorption and interfacial compatibility while maintaining device stability (Figure 2e) [48,52]. Thus, from the very first approaches involving tailored dyes, optimized matching between aqueous media and redox couples, and interfacial engineering, water-based DSSCs have emerged as a promising route toward truly sustainable, safe, and environmentally compatible solar energy devices, highlighting the potential of water as a green electrolyte medium in next-generation photovoltaics. However, stability issues and low photovoltaic power conversion efficiencies (PCEs) have represented major concerns.

**Figure 3:**
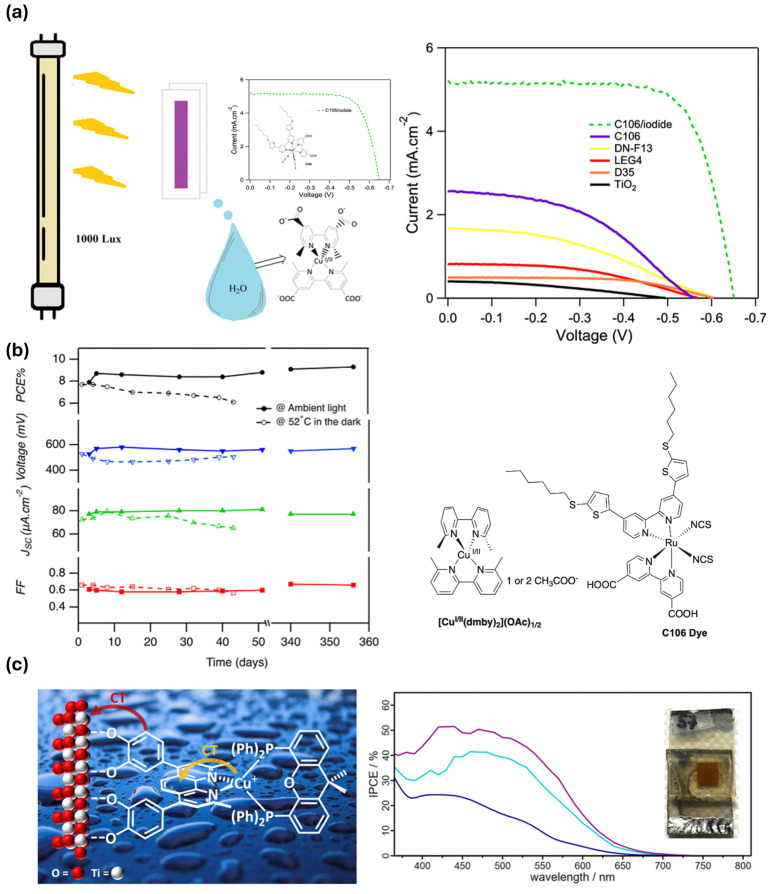
Most representative studies on aqueous DSSCs with Cu(I/II) electrolytes: (**a**) first water-based DSSCs using a Cu(I/II) redox mediator and photocurrent–photovoltage response [61]; (**b**) ligand design of Cu complexes in water-based electrolytes showing a higher long-term operational durability in terms of PCE (black lines), *V*_oc_ (blue lines), *J_s_*_c_ (green lines), FF (red lines). the solid lines refer to devices subjected to ambient light for 1 year, while the dotted lines refer to devices exposed to 52 °C in the dark for 1000 h [62]; (**c**) Cu(I) complexes used as photosensitizers operating in fully aqueous electrolytes [63].

In more recent years, extensive research efforts focused on identifying electrolyte formulations and sensitizers capable of delivering higher PCEs under aqueous conditions [53,54]. Notably, recent reports have demonstrated that carefully engineered aqueous DSSCs can achieve efficiencies exceeding 7% using cationic conductive polymers as counter electrodes, entirely metal-free configurations, or natural dyes such as chlorophylls, underscoring, despite the modest efficiencies (in the range of 1.4–7%) compared with the record PCE of 15.2% for VOC-based DSSCs [55], the potential for fully eco-compatible solar cells [56,57]. In addition to the optimization of dyes, recent studies have also highlighted that specific additives in water-based electrolytes can significantly affect the morphology of the TiO_2_ photoanode. For instance, Gunlazuardi et al. demonstrated that the incorporation of sodium alginate influences the structure of anodized TiO_2_ nanotubes, potentially impacting dye loading, electron injection, and overall device performance [58]. Considering the important advances described above, current research appears to focus primarily on Cu-based redox mediators dissolved in aqueous media. These electrolytes have gained increasing attention as a viable class of aqueous systems for sustainable DSSCs, due to their low toxicity, earth abundance, fast electron transfer kinetics, and tunable coordination chemistry [59]. Unlike traditional I^−^/I_3_^−^ systems, Cu(I/II) complexes offer the possibility of higher open-circuit voltages, thanks to more favourable redox energy levels and reduced corrosiveness, making them particularly appealing for long-term, eco-compatible device operation [60]. A first relevant step forward was reported in 2022, which demonstrated the feasibility of an eco-friendly water-based DSSC for indoor application employing a Cu(I/II) redox couple, highlighting improved safety, reduced environmental impact, and competitive photovoltaic performance in aqueous media (Figure 3a) [61]. Building on this foundation, a more recent study by Ghaddar et al. further advanced the field by addressing the stability bottleneck of aqueous electrolytes, showing that appropriate ligand design and media optimization can dramatically enhance long-term operational durability, positioning aqueous Cu(I/II) systems as one of the most promising candidates for next-generation sustainable DSSCs for indoor application (Figure 3b) [62]. Riding the wave of growing interest in the use of Cu in DSSC architectures, Burmeister et al. demonstrated that copper can also serve as a sensitizer, reporting a DSSC based on Cu(I) complexes with catechol anchoring groups operating in fully aqueous electrolytes, achieving measurable photoconversion efficiency and stable dye attachment. (Figure 3c) [63].

In the interplay with electrolyte engineering, recent work has highlighted that the molecular design of the sensitizer itself can play a decisive role in enabling compatibility with aqueous environments. In this context, we have reported the synthesis of novel dyes featuring a hydroxamic acid anchoring group in place of the conventional cyanoacrylic acid [46]. Hydroxamic functionalities are known to form strong chelating bonds with metal oxide surfaces and to exhibit enhanced stability in water, as demonstrated in photoelectrochemical water-splitting experiments for hydrogen generation. This dye also delivered promising performance in DSSCs employing traditional VOC-based electrolytes, confirming that alternative anchoring strategies can ensure robust attachment to TiO_2_ while maintaining efficient electron injection, paving the way for their application in water-based DSSCs. However, despite the promising results obtained in this field, it is necessary to remember that most water-based devices reported in the literature rely on deionized freshwater. Producing such high-purity water requires additional energy, and freshwater itself constitutes only 2.5% of the Earth’s water resources [64]. This raises significant concerns in regions where water scarcity and drought are critical issues, as it may compete with water resources intended for human consumption. In the pioneering study by Sangiorgi et al., seawater, accounting for 97.5% of global water and naturally rich in ions (e.g., Cl^−^, Na^+^, Mg^2+^, SO_4_^2−^, Ca^2+^, K^+^), thus exhibiting high ionic conductivity (~50 mS cm^−1^ at 20 °C), was tested for the first time as a DSSC electrolyte medium [65]. Both untreated seawater and partially evaporated seawater were investigated to explore how natural ion concentrations and precipitation during evaporation affect electrochemical and photovoltaic performance, with results benchmarked against a deionized water reference electrolyte. Nevertheless, PCEs with seawater-based electrolytes (~0.4%) remain far from being competitive with conventional organic or high-performance aqueous systems [66]. The main photovoltaic parameters and performance metrics are summarized in Table 1.

Although aqueous electrolytes show many benefits, their use still poses challenges in terms of dye efficiency and stability in water. Aqueous DSSCs typically exhibit performance that is currently inferior to conventional VOC-based devices under standard AM 1.5G irradiance. While the current certified record for DSSCs stands at 13.0% according to the latest Solar Cell Efficiency Tables (Version 67) [67], laboratory-scale research has recently pushed the boundaries further, reaching a record PCE of 15.2% and demonstrating long-term operational stability (e.g., over 500 h) [55]. Moreover, regardless of this peak record, state-of-the-art VOC-based DSSCs employing cobalt or copper redox couples have consistently stabilized their average efficiencies between 11% and 13% [3,4], often maintaining 80–90% of their initial performance after 1000–2000 h of continuous light-soaking tests [68,69,70]. This superior performance is primarily attributed to the use of hydrophobic organic solvents that prevent dye desorption and minimize interfacial recombination. Finally, another drawback related to the widespread adoption of water in DSSCs lies in the geopolitical and socio-economic issues, as access to freshwater is unevenly distributed worldwide and is expected to become an increasingly scarce and valuable resource in the future [66]. Consequently, alternative sustainable electrolytes are being actively explored, among which DES-based electrolytes represent a very promising option, as many DESs exhibit low toxicity, high stability, and tunable physicochemical properties suitable for eco-friendly DSSCs.

**Table 1:** Summary of the photovoltaic parameters and performance metrics of the best performing DSSCs in water-based electrolytes.

Redox Couple	Dye	Incident Light Intensity	PCE (%)	*V*_oc_ (V)	*J*_sc_ (mA/cm^2^)	Ref.
I_3_^−^/I^−^ + Triton X	N719	1 sun	5.90	0.81	11.77	[45]
I_3_^−^/I^−^	JK2	1 sun	5.46	0.75	9.60	[48]
Co^(II/III)^(bpy)_3_	MK2	1 sun	5.00	0.67	9.80	[49]
TEMPO/TEMPO^+^	LEG4	1 sun	4.14	0.95	5.78	[50]
I_3_^−^/I^−^ + surfactant	N719	1 sun	3.96	0.68	10.97	[51]
I_3_^−^/I^−^ + buffer solution	V35	1 sun	2.70	0.62	6.65	[52]
I_3_^−^/I^−^	D149	1 sun	6.64	0.69	12.40	[56]
I_3_^−^/I^−^+ eosin Y dye	eosin Y	1 sun	0.06	0.39	0.35	[57]
I^–^/I_3_^–^ + 0.5% Triton X-100 in water	C106	1 sun	2.44	0.65	5.20	[61]
Cu^(I/II)^(dc-dmbpy)_2_Cl_1/2_ + 0.5% Triton X-100	C106	1000 lux	7.20	0.52	0.05	[61]
Cu^I/II^(dmby)_2_ + 2.3 M HNMBI^+^OAc^–^	C106	1 sun	1.05	0.74	2.49	[62]
Cu^I/II^(dmby)_2_ + 3.3 M HNMBI^+^OAc^–^	C106	1000 lux	7.90	0.52	0.081	[62]
Cu based electrolyte + catechol	N719	1 sun	0.46	0.43	1.78	[63]
I^–^/I_3_^–^ + seawater	N719	1 sun	0.37	0.54	1.08	[65]

## 3. DES-Based DSSCs

DESs have emerged over the last fifteen years as one of the most promising alternatives to conventional VOCs or ILs for DSSC electrolytes. The key advantage of DESs lies in their low cost, biodegradability, non-volatility, and facile preparation from readily available components. A DES is typically composed of a hydrogen-bond acceptor (HBA), one of the most common of which is choline chloride (ChCl), and a hydrogen-bond donor (HBD) such as glycerol (Gly), ethylene glycol (EG), urea, or other polyols/acids [42,71]. Although highly promising, the use of DESs as electrolyte media requires careful investigation of their key physicochemical properties for effective application: viscosity, ionic conductivity, thermal stability, and molecular interactions. All these parameters critically affect the performance of DSSCs. Indeed, their tuning enables the design of DES-based electrolytes with improved PCE and sustainability [72].

The first application of DESs as electrolyte media in DSSCs dates back to 2009, when Jhong et al. introduced a ChCl/Gly (1:3) DES diluted with 15 wt% water as a co-solvent in an IL-based electrolyte for DSSCs functionalized with an indoline dye [73]. This pioneering study demonstrated that DESs could successfully replace VOCs in the electrolyte composition while maintaining basic device operation, achieving a PCE of 3.9%. Despite its lower efficiency compared to acetonitrile-based reference cells (4.9%), this work highlighted the potential of DESs as green and low-toxic electrolyte media for DSSCs. In the following years, research focused on optimizing DES compositions to balance viscosity, ionic conductivity, and interfacial compatibility. Hydrophilic DESs such as ChCl/EG and ChCl/urea were tested as electrolyte media, often with controlled amounts of water (typically 20–40 wt%), to improve ion transport [74,75,76,77,78]. These electrolytes using aqueous DESs as media enabled the use of hydrophilic dyes and co-adsorbents tailored to polar media, enhancing dye-sensitizer interactions and reducing aggregation on TiO_2_ surfaces. However, the addition of water to DES-based systems must be carefully balanced. Although water reduces viscosity, excessive content reintroduces the stability issues typical of fully aqueous systems, namely the long-term degradation of the dye–TiO_2_ bond and the potential leaching of the sensitizer [42].

**Figure 4:**
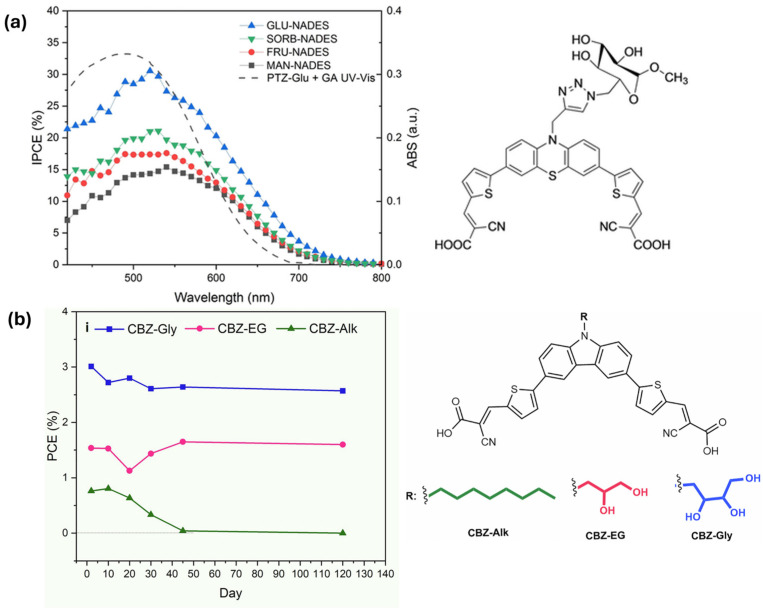
Examples of studies on the structure–property relationships between dyes and DES composition. (**a**) IPCE curves of DSSCs based on new carbazole dyes and NADESs as electrolyte medium [76]; (**b**) structure–activity relationships between carbazole dyes and DES composition and effects on the PCE stability over time [8].

In 2019, we investigated the use of an aqueous ChCl-based DES combined with a phenothiazine dye (PTZ) bearing an oligoethylene glycol chain (PTZ-TEG), which led to improved supramolecular dye organization and, albeit modestly, an enhanced PCE (1.7% at 1 sun and 1.9% at 0.5 sun) compared to DSSCs sensitized by conventional alkyl-functionalized dyes [74]. A year later, we extended the study to a menthol-based hydrophobic eutectic solvent as the device medium [75]. Such a medium allowed the application of traditional hydrophobic DSSC organic sensitizers. Although higher viscosities limited photocurrents compared to VOC-based DSSCs, the photovoltage increased due to reduced charge recombination at the TiO_2_/electrolyte [75]. In more detail, we hypothesized that this result may stem from a more efficient interface interaction, as suggested by electrochemical impedance spectroscopy studies showing greater charge recombination resistance and electron lifetime [79,80]. To further increase the sustainability of the devices, a more recent study by our group explored the use of the so-called “natural deep eutectic solvents” (NADESs) as electrolyte media, combining ChCl with carbohydrates such as glucose, sorbitol, or fructose, often in the presence of 20–40% water (Figure 4a) [76]. These NADESs, in combination with hydrophilic donor-acceptor dyes (e.g., PTZ-Glu, a PTZ dye bearing a terminal glucose-based functionality) and optimized co-adsorbents, demonstrated that synergistic interactions among DES components, dye, and co-adsorbent can significantly enhance charge transport and device performance. Incident photon-to-current efficiency (IPCE) values of up to 30% were recorded across the Vis spectrum.

More recently, our group provided a significant contribution by elucidating the structure–activity relationship between carbazole-based dyes bearing pendants of different polarity and the composition of the DES-based electrolyte. Their study clearly demonstrated that the proper molecular matching between the hydrophilic pendant of the dye and the HBA of the DES is crucial to maximize device performance. In particular, the combination of a ChCl/Gly with a carbazole dye functionalized with glycerol pendants resulted in optimized supramolecular interactions, improved dye organization at the TiO_2_ interface, and enhanced charge-transfer dynamics. Such a rational dye–DES matching strategy enabled record efficiencies for DES-based DSSCs under indoor illumination, reaching up to 9.4% PCE [8]. Notably, the same work also showed that the use of a pure DES as electrolyte media (without any addition of water) significantly improved long-term device stability, underscoring the critical role of water content in DSSCs (detrimental hydrolysis of the dye-TiO_2_ bonding) and highlighting that the absence of water in the electrolyte composition can mitigate degradation pathways while preserving high photovoltaic performance (Figure 4b) [8].

**Figure 5:**
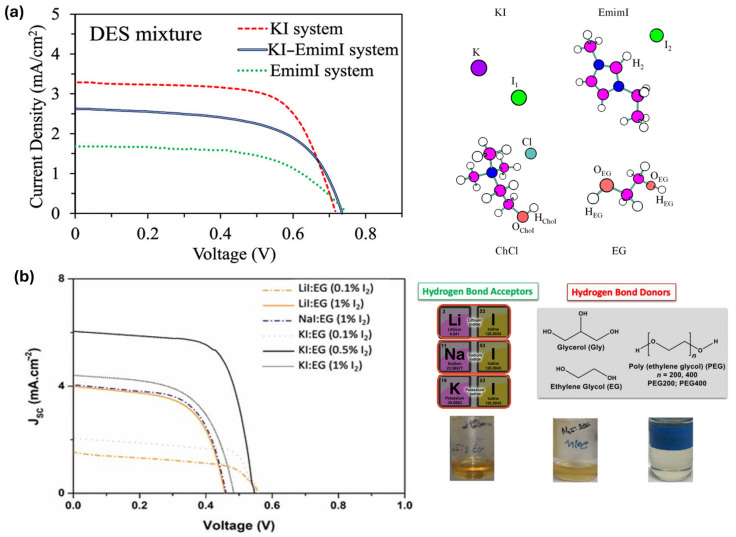
Examples of studies on iodide sources in DES-based electrolytes. (**a**) Current/voltage curves of DES-based electrolytes comparing different iodide sources: small inorganic cations (K^+^, Li^+^) with organic cations (e.g., EMIM^+^). The blue atoms correspond to nitrogen (N) atoms, while the pink atoms correspond to carbon (C) atoms. [77]; (**b**) photovoltaic characteristics of DSSCs employing alkali iodide DESs as alternative electrolyte media [85].

In parallel, attention was given to the choice of iodide sources in DES-based electrolytes. Studies comparing small inorganic (K^+^, Li^+^) with organic cations (e.g., 1-ethyl-3-methylimidazolium, EMIM^+^) revealed that smaller cations can more effectively coordinate with TiO_2_ surfaces, modulating conduction band levels and facilitating electron injection, thereby improving photocurrents (Figure 5a) [77].

DESs have also been investigated as co-solvents in combination with VOCs. These hybrid systems often balance the high conductivity and low viscosity of VOCs with the long-term stability and non-volatility of DESs [81,82]. Building upon the growing interest in DES-based electrolytes, several recent studies have significantly broadened the understanding of how eutectic formulations influence DSSC performance. For example, Nguyen et al. used ChCl/EG mixed with 1-ethyl-3-methylimidazolium tetracyanoborate (EMITCB), achieving PCEs comparable to IL-based cells at lower DES concentrations, while also providing improved photovoltage and electron recombination suppression [83]. This work demonstrated that ChCl-based DESs can be engineered to finely tune viscosity, conductivity, and interfacial recombination dynamics, achieving remarkably stable devices and showing that the ChCl scaffold represents one of the most versatile HBAs for photovoltaic applications.

More recently, we have introduced a novel paradigm for DSSCs based on iodide-based DES-like mixtures acting simultaneously as the electrolyte solvent and sole iodide source, without the need for co-solvents or additional external iodide salts [84]. By combining optimized device architecture with a tailored dye, featuring a strategically positioned hydrophobic chain, the system achieved PCE values up to 4% under standard conditions, along with remarkable stability (~95% retained over more than two months). Even higher efficiencies (up to 8.0%) were obtained under low-light illumination (1200 lux), outperforming many environmentally friendly electrolyte systems such as water-based DSSCs. This approach represents a fully unconventional device configuration and highlights the potential of DES-like media for sustainable, low-cost, and high-performance alternatives.

In parallel, Cruz et al. developed alkali-iodide DESs (KI/EG) in which the redox-active iodide anion is an intrinsic component of the eutectic matrix itself, with PCE up to 2.3% (Figure 5b) [85]. Continuing along this trajectory, a study by Ahmadi et al. further demonstrated that the rational tailoring of DES structures using propylene carbonate (PC) and tetrabutylammonium iodide (TBAI) (PC/TBAI-DES), in combination with the I^−^/I_3_^−^ redox couple, can significantly enhance electron transport and *V*_oc_, bringing DES-based DSSCs closer to the performance levels of conventional VOC-based devices [86]. Collectively, these studies mark a clear evolution from early proof-of-concept DES electrolytes to highly engineered systems where the eutectic matrix is no longer a passive solvent but an active, tunable component enabling improved stability, reduced recombination, and more sustainable device architectures. A summary of the photovoltaic parameters and performance metrics is presented in Table 2.

Additionally, the versatility of DESs extends beyond their role as bulk solvents, as they can also function as effective additives for interfacial engineering. Nguyen et al. explored the possibility of using eutectic mixtures based on ChCl and phenol, demonstrating that such systems can reach efficiencies of nearly 7% when used in binary mixtures, with an increase up to 7.75% of PCE after 1300 h [82]. A few years later, the same group highlighted the ability of ChCl:phenol to improve the *V*_oc_ by approximately 10–40 mV compared to DSSCs without this additive [87]. These phenomena were attributed to shielding effects and optimized charge-transfer processes at the electrode/electrolyte interfaces.

Collectively, these advances position DES-based electrolytes as compelling alternatives to both VOCs and water-based systems, particularly for next-generation indoor and integrated photovoltaic technologies where stability, safety, and sustainability are essential.

However, while DES formulations markedly improve chemical stability, reduce volatility, and enable tunable ionic transport, their intrinsically high viscosity and potential limitations in long-term mechanical robustness may still hinder large-scale device integration. To address these constraints, increasing attention has been directed toward hydrogels and polymer-based quasi-solid electrolytes, which merge the green chemistry benefits of DESs/aqueous systems with enhanced mechanical integrity, leakage suppression, and structural flexibility. These quasi-solid architectures therefore represent a logical and strategic evolution in the development of sustainable DSSCs, bridging high performance with improved durability and practical applicability.

## 4. Bio-Derived Quasi-Solid Electrolytes in DSSCs

**Figure 6:**
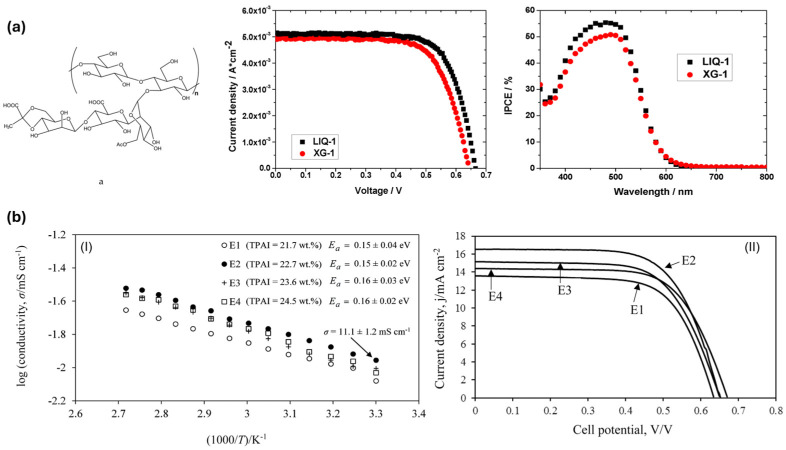
Examples of bio-derived quasi-solid electrolytes in DSSCS. (**a**) *J/V* curves and IPCE of DSSCs fabricated with aqueous electrolytes gelled with xanthan gum [91]; (**b**) DSSCs fabricated using phthaloylchitosan gel polymer, (I) T dependence of the electrical conductivity for electrolytes different TPAI contents, (II) *J/V* curves of DSSCs fabricated using such electrolytes [98].

As DSSC research increasingly emphasizes sustainability and long-term operational stability, solid-like electrolytes based on polymeric and bio-derived matrices have gained significant attention. These systems aim to replace VOC-based formulations with structurally confined, environmentally benign alternatives capable of ensuring both device safety and adequate ion transport. The shift away from volatile organic solvents initially stimulated the development of synthetic polymer gels, such as poly (β-hydroxyethyl methacrylate)-based systems [88], poly (acrylonitrile-co-vinyl acetate) (PAN-VA) quasi-solid gel electrolyte media [89], and poly (vinylidene fluoride (PVDF)-based electrolytes [90], which demonstrated that quasi-solid electrolytes could approach or even rival their liquid counterparts in terms of efficiency, while offering superior thermal and mechanical stability. These early high-performance gel systems, in some cases exceeding 7–8% PCE, established the technological feasibility of leakage-free and mechanically robust DSSCs and laid the groundwork for the subsequent integration of greener polymer matrices. However, in terms of sustainability, recent approaches have focused on the use of bio-derived and water-processable polymers, aiming to combine quasi-solid formulations with environmental compatibility. Among natural polysaccharides, xanthan gum has emerged as a pioneering material. Early studies demonstrated that xanthan-based thixotropic hydrogels could effectively infiltrate the mesoporous TiO_2_ network, maintaining continuous ion diffusion pathways while significantly suppressing solvent evaporation compared to liquid formulations [91,92]. Subsequent systematic investigations by Galliano et al. established a fully aqueous xanthan-gum gel matrix employing the I^−^/I_3_^−^ and Co(II/III) redox couple, achieving power conversion efficiencies in the 2.7–4% range and V_oc_ up to ~850 mV with Co-based mediators. Importantly, operational stability extended beyond 1200 h, confirming that biopolymer confinement can dramatically improve long-term durability without severely compromising ionic conductivity (Figure 6a) [91,93]. In the same years, research broadened to other renewable matrices. Polysaccharide-based electrolytes demonstrated that natural polymer networks can stabilize the electrolyte–electrode interface while maintaining acceptable ion mobility [94,95,96]. Chitosan-based gel matrices incorporating iodide salts further expanded the polysaccharide toolbox, delivering moderate but stable efficiencies and highlighting the versatility of bio-based ionic pathways (Figure 6b) [97,98,99].

**Figure 7:**
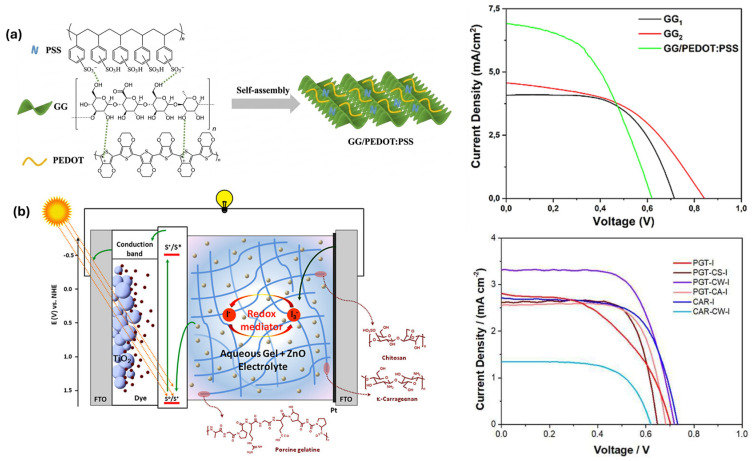
Examples of studies on natural polymer-based electrolyte media in DSSCs: (**a**) interactions between gellan gum and PEDOT:PSS in the DSSC electrolyte and the *J/V* curves of the devices [101]; (**b**) study on the implementation of CDCA directly within natural polymer-based hydrogels to control supramolecular interactions and improve dye organization when using an electrolyte matrix [102].

More recently, galactomannan-derived hydrogels combined with zinc salts or ZnO nanostructures have enabled quasi-solid aqueous DSSC fabrication with *V*_oc_ around 750 mV, enhanced mechanical robustness, and optical transparency suitable for semi-transparent or building-integrated photovoltaics [100]. More recent efforts have explored hybrid gel architectures combining biopolymers with electronically conductive components. In this direction, in the work of Unlu et al. [101], gellan gum/PEDOT:PSS gel electrolytes incorporating a conductive polymer within a natural hydrogel matrix demonstrate the possibility to enhance both mechanical integrity and interfacial charge transport in quasi-solid DSSCs (Figure 7a) [101]. By leveraging the structural properties of gellan gum together with the electrical functionality of PEDOT:PSS, the resulting gel-based electrolyte exhibited improved stability and competitive photovoltaic performance. At the same time, hybridization strategies have further enriched hydrogel functionality. The incorporation of inorganic nanoparticles within natural polymer matrices such as Al_2_O_3_ or ZnO dispersed in polysaccharide gels has been shown to improve ionic conductivity and interfacial stability through nano-engineering of the polymer network. Conductive polymer integration represents another important step forward: a recent study on gellan gum/PEDOT:PSS gel matrices demonstrated that blending biopolymers with electronically conductive additives can simultaneously enhance mechanical strength and charge-transport properties of the electrolytes, offering a promising route toward mechanically resilient quasi-solid DSSCs. Parallel advances in interfacial engineering have also emerged, exemplified by Casoli et al. [102] in the 2025 implementation of chenodeoxycholic acid (CDCA) directly within natural polymer-based hydrogels, where controlled supramolecular interactions improved dye organization, reduced recombination, and enhanced low-light performance, an essential requirement for indoor photovoltaics [102]. Beyond photovoltaic conversion alone, hydrogel-based electrolytes are increasingly being integrated into multifunctional platforms. For instance, Domenici et al. in 2025 reported a xanthan-gum-based hydrogel DSSC coupled with a supercapacitor, achieving an indoor light conversion and storage efficiency of 1.45%, thereby illustrating the compatibility of bio-derived quasi-solid electrolytes with integrated energy-harvesting and storage systems [103]. A summary of the photovoltaic parameters and performance metrics is presented in Table 3.

Overall, the evolution of polymer-based quasi-solid electrolytes from early synthetic high-performance gels to bio-derived, multifunctional hydrogel architectures reflects a clear convergence between performance optimization and sustainability. By combining biodegradable matrices, aqueous or environmentally benign redox mediators, improved mechanical integrity, and compatibility with low-intensity illumination, it is possible to obtain robust and versatile DSSCs for safe, durable, and integrable photovoltaic technologies tailored to indoor and ambient-energy applications.

## 5. Conclusions and Outlook

Sustainable DSSCs have made remarkable progress over the past decade, driven largely by innovations in electrolyte design. Traditional VOC-based electrolytes, while enabling high efficiencies, suffer from volatility, toxicity, and stability issues, limiting their practical applicability, particularly for indoor and integrated photovoltaics. In response, water-based electrolytes emerge as an important step toward greener DSSCs, offering low toxicity, low cost, and environmental compatibility. Advances in hydrophilic dyes, interface engineering, and alternative redox couples including cobalt-, copper-, and organic-based mediators have contributed to improved stability and performance, with indoor PCE exceeding 7% in optimized systems. Building on these developments, the use of DESs has attracted increasing attention as versatile, tunable, low-cost, abundant, and sustainable alternatives, particularly in contexts where freshwater may represent a critical issue. DES-based electrolytes combine low toxicity, biodegradability, and enhanced thermal stability with high chemical robustness. Several studies have highlighted their potential to improve device lifetime, enhanced ion solubility, and top-performing photovoltage values, particularly under low-intensity indoor illumination. Nevertheless, high viscosity and mass transport limitations remain challenges that must be balanced with their environmental benefits. Finally, biopolymer-based electrolytes offer long-term stability, adequate ionic mobility, and facile integration into flexible or semi-transparent devices. In addition, these systems may enable multifunctional approaches, such as simultaneous energy storage or enhanced *V*_oc_ through nanostructured additives. While ionic mobility and temperature tolerance remain areas for optimization, the use of hydrogels as electrolyte matrices exemplifies the potential to combine performance, sustainability, and safety in a single platform.

Recent studies have further explored the use of DESs beyond DSSCs, including their application in dye-sensitized photocatalytic hydrogen production, where both hydrophobic and hydrophilic DES mixtures have shown promising performance compared with conventional aqueous systems [104]. These findings further support the broader interest in DESs for sustainable solar-energy-conversion technologies.

Overall, recent research is converging toward safer and more sustainable DSSCs optimized for indoor and integrated applications, where safety requirements are more stringent, while at the same time achieving high PCE values that are now comparable, or event higher, than those of traditional systems based on toxic, volatile, and hazardous media. Future research is likely to focus on hybrid strategies that combine the best features of aqueous, DES, and hydrogel-based electrolytes: low toxicity, tunable redox chemistry, robust ion transport, and mechanical stability. Continued progress in addressing challenges related to viscosity, interface engineering, and long-term durability will be necessary to assess the large-scale applicability of these systems in low-light photovoltaics, building-integrated photovoltaics, and smart energy-harvesting technologies.

## Figures and Tables

**Table 2:** Summary of the photovoltaic parameters and performance metrics of the best performing DSSCs in DES-based electrolytes for solar energy conversion.

Redox Couple	Dye	DES	Incident Light Intensity	PCE (%)	*V*_oc_ (V)	*J*_sc_ (mA/cm^2^)	Ref.
I^−^/I_3_^−^	D149	ChCl:Gly (1:3)	1 sun	3.88	0.55	12.00	[73]
I^−^/I_3_^−^	PTZ-8	ChCl:Gly (1:2)	1 sun	2.50	0.48	7.30	[74]
I^−^/I_3_^−^	PTZ-ALK	DL-menthol:acetic acid (1:1)	1 sun	2.50	0.58	6.60	[75]
I^−^/I_3_^−^	PTZ-GLU	ChCl:D-glucose (1:2)	1 sun	1.40	0.53	4	[76]
I^−^/I_3_^−^	N719	ChCl:EG (1:2)	1 sun	1.60	0.72	3.28	[77]
I^−^/I_3_^−^	N719	ChCl:guanidinium thiocyanate (1:1)	1 sun	1.72	0.58	4.66	[78]
I^−^/I_3_^−^	N719	ChCl:3-phenylpropionic acid (1:1) + ethanol	1 sun	1.70	0.67	5.07	[81]
I^−^/I_3_^−^	N719	ChCl:phenol (1:2)	1 sun	6.92	0.71	14.44	[82]
I^−^/I_3_^−^	N719	ChCl:EG (1:2)	1 sun	5.11	0.66	9.9	[83]
I^−^/I_3_^−^	TPA-TTh-C_6_	ChI:EG (1:2)	1 sun	3.8	0.66	8.2	[84]
I^−^/I_3_^−^	TPA-TTh-C_6_	ChI:EG (1:2)	1200 lux	8.00	0.43	10.3	[84]
0.5 mol% I_2_	N719	KI:glycerol (1:5)	1 sun	2.30	0.54	5.26	[85]
I^−^/I_3_^−^	N719	(0.10 M) propylene carbonate (PC):TBAI (8:1)	1 sun	10.04	0.78	21.94	[86]

**Table 3:** Summary of the photovoltaic parameters and performance metrics of the best performing DSSCs in bio-derived quasi-solid electrolytes.

Redox Couple	Dye	Organic Matrix	Incident Light Intensity	PCE (%)	*V*_oc_ (V)	*J*_sc_ (mA/cm^2^)	Ref.
I^−^/I_3_^−^	N3	PHEMA ^a^ (organogel)	1 sun	7.46	0.69	14.30	[88]
I^−^/I_3_^−^	N719	PAN-VA (polymer gel)	1 sun	6.43	0.72	14.85	[89]
I^−^/I_3_^−^	N719	M-KL/PVDF-HFP ^b^	1 sun	7.48	0.81	13.51	[90]
I^−^/I_3_^−^	D131	xanthan gum	1 sun	2.70	0.64	4.95	[91]
I^−^/I_3_^−^	TG6	xanthan gum	1 sun	4.78	0.65	9.49	[92]
Co(bpy)^2+/3+^	MK2	xanthan gum	1 sun	4.47	0.79	7.52	[93]
I^−^/I_3_^−^	-	agarose polymer	1 sun	4.14	0.60	8.24	[96]
I^−^/I_3_^−^	N719	chitosan (+ICPs/ILs)	1 sun	0.06	0.33	8.80	[97]
I^−^/I_3_^−^	N3	phthaloyl-chitosan	1 sun	9.47	0.71	19.61	[98]
I^−^/I_3_^−^	N3	phthaloyl-chitosan	1 sun	3.57	0.51	2.46	[99]
I^−^/I_3_^−^	D131	galactomannan + zinc	1 sun	1.75	0.72	3.53	[100]
I^−^/I_3_^−^	N719	gellan gum/+PEDOT: PSS	1 sun	2.01	0.62	6.92	[101]
I^−^/I_3_^−^	D131	PGT-CW ^c^	1 sun	1.56	0.71	3.27	[102]
I^−^/I_3_^−^	D131	PGT-CS ^d^	1200 lux	3.18	0.58	0.032	[102]
I^−^/I_3_^−^	D149	xanthan gum	1 sun	1.24	0.52	3.80	[103]
I^−^/I_3_^−^	D149	xanthan gum	1000 lux	3.51	0.38	0.037	[103]

^a^ Poly (2-hydroxyethyl methacrylate). ^b^ Modified kaolin/poli (vinilden floruro-co-esafluoropropilene). ^c^ Porcine gelatin, CDCA. ^d^ Porcine gelatin–chitosan.

## Data Availability

No new data were created or analyzed in this study.
